# Tuberculosis and Pulmonary Phthisis

**Published:** 1868-10

**Authors:** 


### Miscellaneous.

### Tuberculosis and Pulmonary Phthisis.—A Critical Review.

[CONCLUDED.]

The doctrine of the caseous pneumonia of epithelial phthisis, regarded as the distinct disease of tuberculosis, is not, as is seen, solidly founded upon pathological anatomy, which leaves, as M. Ch. Bouchard says, doubts impossible to remove.

Does this doctrine find a more solid support from clinical experience? We shall now examine this question.

Since I can use the right of the critic, I shall take the liberty of immediately bringing up a contradiction which escaped Niemeyer. In most cases, he says, the three forms of phthisis can be distinguished from each other *almost with certitude;* and a few lines further on, doubtless regretting having said so much, he adds, we shall try to draw the picture of the three principal forms of pulmonary phthisis. M. Bouchard considers the differential diagnosis of simple caseous pneumonia and of caseous pneumonia complicated with tuberculosis as surrounded with almost insurmountable difficulties.

Whatever it may be, let us, however, see upon what signs Niemeyer bases a diagnosis.

1.—Great frequency of the respiratory movements without augmentation of the souffle, and dullness, in a patient who has had for some time the signs of pulmonary induration, may cause us to suspect that a tuberculosis has arisen to complicate an already existing phthisis.

2.—Pains in the chest and through the shoulders more frequently accompany the pneumonic processes than the tuberculosis.

3.—If cough and expectoration have preceded fever and loss of flesh, it is a pneumonic process we have to contend with; if the contrary, then we should regard it as a tuberculosis phthisis.

4.—An intimate mingling of blood with the mucus and muco-purulent sputa constitutes a sign of the pneumonic process.

5.—A persistent cough, continuing a long time, with scanty expectoration, is a sign of tuberculosis.

6.—A hoarse cough is one of the particular signs of tubercular phthisis, or of a complication by tuberculosis of a pulmonary phthisis, due primarily to destructive inflammatory processes.

7.—The type of the fever in simple pneumonic process is remittent; the fever tends towards the continued type in tubercular phthisis and in phthisis complicated with tuberculosis. In other words, in simple caseous pneumonia the temperature in the morning varies little from the normal standard, and rises in the night to 1°, 1°.5 to 2°, while in tubercular phthisis the temperature keeps up above the normal state, sometimes reaching 41°, and does not fall sensibly towards morning. (Recherches sur la température dans la phthisie pulmonaire, par Sidney Ringer, *Archives de Med.*, 1866.)

8.—In tuberculosis, the loss of flesh and the impoverishment of the blood are much more rapid than in caseous phthisis.

9.—A shrill sound, not flat, tympanitic, limited to the summit and coinciding with a diminution in the fullness of the respiratory movements, should lead us to suppose a tuberculosis.

10.—Cavernous sounds, finally, are not produced in pure tuberculous consumptions, and most large caverns *should be considered* as due to the breaking down of the tissues affected with caseous infiltration.

When we consider that, according to Niemeyer, the coëxistence of tubercles and inflammatory caseous masses is very frequent, that granulations, as he always allows, may be developed in the lungs in so latent a manner that they cannot be diagnosticated, where is the physician who, in similar conditions, with such slight differential symptoms, would dare to give a diagnosis, that is, would positively state that a patient is or is not affected with tuberculosis?

If, under a like condition, a vain scientific curiosity only is to be satisfied, I shall not insist upon it, but if we accept the views of the Professor of Tubingen, that upon the precision in diagnosis evidently depends upon the gravity of the prognosis, then it is of the greatest importance that the problem should be solved. If the patient is attacked with a simple caseous pneumonia, it is not impossible to cure it, while if it is a tuberculosis, either primary or consecutive, then treatment is positively of no avail. Therefore, I repeat, the consequences depending upon the diagnosis are so serious that to establish it we require more marked and better defined signs than those indicated by Niemeyer, which he is far from considering as being always sufficient.

To distinguish tuberculosis from caseous phthisis, it has been thought that a criterion has also been found in the duration of the disease, by laying down as a principle that tuberculization is always acute. "It destroys," says M. Bouchard, "rapidly, like acute diseases; when it is prolonged, the new attacks reproduce acute manifestations, and if its total duration attains six or seven months, there is nothing established which shows that it can pursue a slow or gradual course like common consumption."

We can, in fact, regarding it from a certain point of view, say there is no chronic tuberculization, for in rapid consumption as in slow consumption, each new irruption of granulations produces acute symptoms, which are more or less intense, destroying the patient or gradually wearing him out.

In both cases the phenomena are the same; they only differ in their degree of intensity.

To demonstrate the relation between the two diseases, do we not see, every day, patients who present, as the effect of an irruption of granulations, acute symptoms, which would seem enough to carry them off, but who apparently recover, and the disease pursues its fatal course, with all the alluring signs of a chronic affection.

Such was precisely the case of the patient of M. Colin, to whom M. Bouchard alludes, and whom he considers as having had primarily an hæmoptysis and pneumonia; a patient in whom all the symptoms of an acute phthisis were rapidly developed, and which ended by becoming chronic.

Admitting that any form of pneumonia (fibrinous or catarrhal) can terminate, under certain conditions, by caseous infiltration, that a bronchial catarrh can produce catarrhal pneumonia by the propagation of the disease to the alveoli, and pulmonary phthisis by caseous transformation and the consecutive breaking down of the inflammatory products, Niemeyer naturally returns to the theory of a *neglected cold*, which we thought was buried forever.

In this, however, he approaches the opinions of Broussais, who also admits that all pulmonary inflammation can degenerate into phthisis, but into true tuberculous phthisis, tubercle being, according to him, the most ordinary result of these inflammations when they persist beyond their habitual term. (Histoire des phlegmasies chroniques, t. iii, p. 216.)

The first thing, the interpretation of which immediately embarrasses the anthor, is the persistence of a bronchial catarrh, limited to the summits of the lungs without the previous existence of granulations. According to him, this catarrh is not, as is generally believed, the evident sign of a commencing pulmonary tuberculosis, but very certainly a sign which announces that the patient is threatened with phthisis. As death does not ordinarily occur at this period of the disease, Niemeyer can hardly call pathological anatomy to his aid to justify his assertion, and clinical experience does not offer any greater resources.

If tubercle can exist in the latent state, and if bronchitis, when present, is only the indication of it, and M. Villemin agreeing upon this point with Laennec, Louis, Andral and Fournet, is there not, then, reason for regarding the bronchitis which is then induced by the slightest causes, as the effect in reality of the tuberculous neoplasm?

Again, in persons affected with bronchitis the signs of phthisis are concealed by those of inflammation of the bronchi, and only appear when the diminution of the local symptoms of bronchitis reveals those of tuberculization, which are on that account none the less primitive.

The same remarks are applicable to chronic inflammations of the lungs and pleura, and according as these complications are predominant, they control the pathological condition, even completely conceal, at times, the presence of tubercles, and cause the primordial lesion, of which they are only the effect, to pass unperceived. It is this form of disease which Broussais proposed to call tuberculous pneumonia, in contradisdinction to pneumonic phthisis, that is to say, that in which tubercles manifestly preceded the pulmonary inflammation.

It does not follow from this that tuberculization cannot be developed in a person some time affected with bronchitis, pneumonia or simple pleurisy; but it seems to me necessary to recognize some relation of cause and effect between these facts. We may say, with M. Pidoux, that tubercular and catarrhal diseases pursue two parallel lines, and however prolonged they may be supposed to be, these two lines may never meet. When we treat of the nature of tubercle and tuberculosis, we shall have occasion to speak of this point more in detail.

Pulmonary tuberculization, as all admit, progresses by successive irruptions of granulations, which excite about them more or less severe inflammation. This normal course of the disease, it appears to me, satisfactorily explains the various phenomena which Niemeyer, in common with all observers, has remarked in the course of pulmonary phthisis. After each irruption of granulations, there are present for some time acute symptoms, the progress of the disease is accelerated, then comes a period of comparative calm up to the breaking out of new granulations; and I find it entirely useless, on this account, to say with Lebert, that a patient can be consumptive to-day, may not be so in three months, and may be so again in six months.

Primitive tuberculosis, at the beginning of phthisis, being the exception, according to Niemeyer, in order to explain the frequency of hæmoptysis at this period of the disease, he admits then that the hæmorrhage and the phthisis come *simply* from a common source, that is to say, from a double predisposition of the patient to hæmorrhage and to phthisis. Hæmorrhage, according to the Professor of Tubingen, can precede phthisis, and induce chronic inflammations of the lungs, which are followed by destruction of the latter. If I did not hesitate to surpass the limits of a critical review, which should always be courteous, I should retort upon Niemeyer the reproach he addresses to Laennec of having made an unfortunate application of the principle, "*Post hoc, ergo propter hoc.*"

In most cases, an hæmoptysis is followed by a more or less severe irritation of the lung or pleura; this fact is incontestable, and can be easily explained if we admit the doctrine of Laennec—this aggravation is nothing more than the index of a new irruption. Hæmoptysis, says M. Villemin, can only be the result of pulmonary congestion at the commencement of the evolution of the process, and of the obliteration of the vessels in the neighborhood of the nodules, whence increase of intra-vascular pressure, then rupture of capillaries, and bloody extravasation. Is not this interpretation of the facts, which clinical experience, moreover, infinitely more satisfactory than the hæmorrhagic diathesis invoked by Niemeyer?

It seems to me that a definite conclusion may be drawn from this long discussion—that pathological anatomy does not furnish proofs sufficient to establish the independence of caseous pneumonia and tuberculosis; that clinical experience is even still more powerless in demonstrating this quality of pulmonary phthisis.

If we admit that tuberculous granulation preëxists, that it is always the primordial lesion, then all is joined together, all is united. If, on the contrary, this great fact is not admitted, everything becomes confused, uncertain, and as there can be but one true theory, the multiplicity of doctrines which we have seen successively arise, can only have the effect of exciting doubt in the minds of those who would believe.

For instance, catarrhal phthisis—caseous pneumonia of Niemeyer, caseous phthisis of M. Coursieres, epithelial phthisis of M. Feltz and of M. Chatin, tuberculiform phthisis of M. Hirtz, tuberculide of M. Sorel, disseminated chronic pneumonia of Lebert, etc.; this phthisis, I say, which does not want for names, is even to the best informed nothing else than demonstrated.

For my part, I am convinced that the doctrine of Laennec will come out triumphant from all attacks, that modern researches will only give to it a still more brilliant confirmation, and, finally, that M. Villemin was inspired when he proclaimed anew that every pulmonary consumption was the result of the evolution of tubercles.

But there is a disease also characterized anatomically by granulations disseminated in different organs, and especially in the serous membranes, which, from its peculiar symptoms and its generally rapid course, seems at first to merit a distinct place in the nosological table—I speak of acute tuberculization.

M. Empis, adopting entirely this view, has made a special disease of it, and has called it granulie.

Other authors, returning to the views of Bayle, have also desired to make a particular disease of that phthisis which is characterized by the rapid development of miliary granulation in the lung (phthisie granuleuse miliare.)

But if we closely examine these facts, we shall see that these distinctions have no foundation.

One thing seems to me to be immediately established, that it is not possible to consider as two distinct diseases granular pulmonary phthisis and acute tuberculization. It seems to me that this granular phthisis rather serves as a uniting link between chronic phthisis and acute tuberculization, and for two reasons: first, because it is extremely rare not to meet granulations in other organs at the same time that they are found in the lungs; and again, because if the pulmonary disease has continued for some time, we find at the autopsy that a certain number of gray granulations have already undergone fatty transformation.

M. Colin has very clearly indicated this latter fact in his clinical studies, and the third observation of acute tuberculization which he reports can leave no doubt upon the subject.

But when the granulations are general, when they invade the meninges, the pleuræ, the peritoneum, the pericardium, etc., the disease no longer presents the same aspects.

Granulation, always identical, produces different effects according to the organ in which they are developed, and there is nothing in this fact which can justify a fundamental distinction.

Age, in particular, exercises very marked influence upon the degree of generalization of granulations, and Lebert observed with reason that tubercles are so much the more abundant and are disseminated through a greater number of organs, the younger the person and the nearer he is to infancy.

When at the autopsy no softened granulations are found, it is, as M. Peter remarks, because the rapid progress of the disease has not allowed the fatty transformations to take place.

Consequently, according as this or that organ is the seat of the disease, according to the number of organs affected, according to the abundance of the granular irruption, according, finally, to the conditions special to each individual, the symptoms, the progress, the termination even of the disease differs, but the anatomical lesion is always the same, and continues to characterize the affection.

Examples of diseases identical in their nature and different in their clinical aspect, are frequent in pathology.

The lobar pneumonia of the infant, the acute pneumonia in the adult, and the insidious pneumonia of the aged, although very different when regarded from a clinical point of view, are nevertheless one and the same disease.

A varioloid and a confluent variola, differing as they do in seriousness, are still the result of one and the same cause. An acute cancer does not differ as to its nature from a cancer which pursues slowly and fatally its destructive course.

I do not intend to insist upon this point; it is only necessary to present the question in such terms, in order to have a unanimous response from all clinical teachers.

Not content with complicating facts, names have been completely mystified. The granulie of Empis is the tuberculization of Virchow; the tuberculization of Empis is the caseous pneumonia of the Germans. Galloping consumption was admitted as designating the common, vulgar phthisis, having a rapid course; and acute consumption, as designating pulmonary granular phthisis, until Trousseau, and after him M. Jaccoud, in his notes to Graves's Clinical Lectures, overturned completely this nomenclature.

To avoid all confusion, it seems to me that it would be much simpler to admit a pulmonary tuberculization, or, if you please, a *chronic pneumophymia*, (common, vulgar consumption,) a *subacute pneumophymia* (common phthisis running a rapid course,) and an acute phthisis (granular phthisis,) reserving the expression of *acute tuberculization* for those cases where the granulations are more or less general.

In resuming—the doctrine of the unity of tubercular diseases is not rendered hypothetical by clinical experience nor by pathological anatomy, and in terminating I shall repeat, with the authors of the Compendium, that the only true manner of philosophically regarding pulmonary phthisis is that which consists in considering it as the localization in the lung of a general affection called *tuberculization*, which can be localized in different organs.

I have designedly left completely untouched the question as to the nature of tubercle and tuberculosis. This will be the subject of a second article.

